# Recovery time variation during sprint interval training impacts amateur soccer players adaptations – a pilot study

**DOI:** 10.5114/biolsport.2023.116008

**Published:** 2022-06-01

**Authors:** Gürkan Diker, Abdulkerim Darendeli, Karim Chamari, Alexandre Dellal, Sürhat Müniroğlu, Sadi Ön, Hüseyin Özkamçı

**Affiliations:** 1 Department of Physical Education Teaching, Faculty of Sports Sciences, Sivas Cumhuriyet University, Sivas, Turkey; 2 Aspetar, Orthopaedic and Sports Medicine Hospital, FIFA Medical Centre of Excellence, Doha, Qatar; 3 ISSEP Ksar-Said, La Manouba University, Tunisia; 4 FIFA Medical Excellence Centre, Santy Orthopedicae Clinical, Sport Science and Research Department, Lyon, France; 5 Department of Coaching Education, Faculty of Sports Sciences, Ankara University, Ankara, Turkey; 6 Department of Coaching Education, Collage of Physical Education and Sports, Ahi Evran University, Kırşehir, Turkey; 7 Department of Coaching Education, Faculty of Sports Sciences, Dokuz Eylül University, İzmir, Turkey

**Keywords:** Football, Maximal oxygen capacity, Endurance, Exercise testing, Graded treadmill test

## Abstract

The objective of the present study was to investigate the selected performance adaptations of amateur soccer players to 2 different running-based sprint interval training (SIT) protocols with different recovery intervals and work-rest ratios (1:5 & 1:1). Twenty-three subjects (age 21.4 ± 1.1 years; height 175.4 ± 4.7 cm; body mass 69 ± 6.4 kg) participated in the study. Before the 6-weeks training period, participants completed 3-weeks of low-intensity training preparation. Subsequently, the pre-tests (anthropometric measurements, repeated sprint test [12 × 20-m with 30-s recovery intervals], Yo-Yo_IRT1_ & Yo-Yo_IRT2_ and treadmill VO_2max_ test) were conducted. Thereafter, participants were randomly divided into 3 sub-groups (1 – SIT with 150 s recovery intervals [SIT150, n = 8]; 2 – SIT with 30 s recovery intervals [SIT30, n = 7]; and 3 – control group [CG, n = 8]). SIT150 and SIT30 training groups completed sprint interval training (2-days/week; 30-s all-out running, 6–10 repetition with 150 s recovery intervals for SIT150 and 30 s for SIT30 groups, respectively), a soccer match (1-day) and routine soccer training (3-days) per week. The CG attended only routine training sessions and the soccer-match (4-days). The study experiments and the trainings were conducted during off-season. Yo-Yo_IRT1_, Yo-Yo_IRT2_, and VO_2max_ were significantly improved both in SIT30 and SIT150 (p < 0.05) groups. Yo-Yo_IRT1_ and VO_2max_ were also significantly improved in CG (p < 0.05). Both the SIT150 and SIT30 training were shown to improve Yo-Yo_IRT1_, Yo-Yo_IRT2_ and VO_2max_ performance compared to the control group, nevertheless, SIT150 was more efficient in improving the Yo-Yo_IRT1_, Yo-Yo_IRT2_ than SIT30. The authors of this study suggest using SIT150 to induce more effective performance outputs in amateur soccer players.

## INTRODUCTION

It is known that short-lasting maximal and near-maximal efforts with varying recovery intervals represent essential characteristics of the physical activity within soccer games [1, 2]. Soccer players perform between 10496 m (central defender) and 11779 m (axial midfielder) including 193.6 m – 278.2 m runs > 24.1 km · h^−1^ [3]. 243–364 changes of directions < 90° and 1000–1400 actions including 220 at high intensity (HI) [4]. Consequently, it is imperative to have sufficient physical capacity for soccer players to be able to cope with fatigue through the intense periods of the game where the anaerobic mechanisms prevail, especially very high intensity (VHI) activities [5, 6, 7, 8]. The need to produce HI and VHI activities all across soccer game encourages coaches and sports scientists to utilize different training methods based on HI efforts. To increase the capacity of the players to produce repeated HI and VHI activities, short-lasting high-intensity exercises such as sprint interval training (SIT) can be used in training.

SIT generally utilize three to seven 10–30 s all-out sprint bouts with various recovery periods in between [9, 10, 11]. Soccer players spend more time in HI exercise domain during SIT [9]. which is one of the most effective means to improve running performance. However, its effectiveness depends on recovery, positional roles and individual differences among soccer players. Lloyd Jones et al., [12] compared two SIT protocols with matched work-rest ratio and total sprint duration (the first group: 6 s all-out effort, 20 reps with 48 s rest; and the second group: 30 s all-out effort, 4 reps with 4 min rest; for two weeks) and investigated their adaptations on VO_2max_ performance. Authors reported that both 6 and 30 s all-out SIT training elicited similar changes in performance. Another study, comparing two 10 s (with 2 and 4 recovery intervals min) and a 30 s SIT exercises, showed that all three of the SIT protocols increased oxidative and anaerobic performance [13].

Thomassen et al. [14] compared two groups of SIT. They reported that group-1 (6–8 reps of 20-s all-out running, with 120-s of passive recovery in-between) and group-2 (same efforts, with 40-s of passive recovery intervals) training improved Yo-Yo_IRT2_ (intermittent recovery test) performance by 10% and 4% respectively. Similar studies also support that high-intensity interval training enhances aerobic and anaerobic performances [15, 16, 17, 18]. Considering various training options, this topic needs further investigations. Indeed, the changes in duration, intensity, number of sets and recovery intervals of training provide different metabolic, cardiovascular and neuromuscular improvements [16, 17, 18, 19]. Furthermore, there is a lack of information about how amateur soccer players adapt to widely used SIT protocols with different work-rest ratio. Therefore, the aim of the present study was to investigate the selected performance adaptations of amateur soccer players to 2 different running-based SIT protocols with different recovery intervals and work-rest ratios (1:5 & 1:1). We hypothesized that SIT150 protocol would improve Yo-Yo_IRT1_, Yo-Yo_IRT2_ and VO_2max_ relatively better compared to SIT30.

## MATERIALS AND METHODS

### Subjects

A total sample size of 21 was required to detect an effect size of 0.4 with α = 0.05 and 80% power. Initially, twenty-four male soccer players competing in an amateur soccer team with at least 5-year active soccer exercise background volunteered in this study. The participants were randomly subdivided into 3 sub-groups (1 – SIT150: n = 8, 30-s all-out running, 6–10 repetition with 150 s recovery intervals; 2 – SIT30: n = 7, 30-s all-out running, 6–10 repetition with 30 s recovery intervals; and 3 – CG: n = 8, control group). During the training 1 participant (from SIT30 group) was injured and consequently excluded from the study, leaving a total of 23 participants (SIT150: mean ± SD; age 21.1 ± 1.3 years, height 176.6 ± 4.7 cm, body mass 71 ± 5.9 kg; SIT30: age 21.4 ± 1.1 years, height 177 ± 5.4 cm, body mass 69.4 ± 7.3 kg; CG: age 21.4 ± 1.1 years, height 173 ± 3.5 cm, body mass 66.7 ± 6) (Table 1.). This study was approved by the Ankara University Faculty of Medicine Ethics Committee (ID: 01-13-17) before the data collection process and was conducted in accordance with the standards of ethics. Prior to signing informed consent form, all subjects were informed about the aim, methodology, possible risks, and benefits of the investigation.

**TABLE 1:** Subject characteristics.

	SIT150	S30	CG
Age (years)	21.1 ± 1.3	21.2 ± 0.9	22.1 ± 1.2
Height (cm)	176.6 ± 4.7	177.0 ± 5.4	173.0 ± 3.5
Body Mass (kg)	71.0 ± 5.9	69.4 ± 7.3	66.7 ± 6.1

### Experimental Approach

**FIG. 1:**
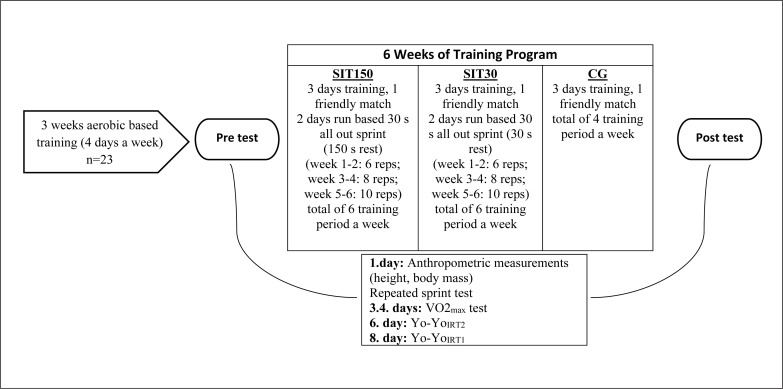
Depiction of the study design.

Pre- and post-training tests including anthropometric evaluations, treadmill VO_2max_ test, Yo-Yo_IRT1_, Yo-Yo_IRT2_ and repeated sprint ability (RSA) tests were performed to examine the performance adaptations induced by 2 different speed endurance training (Figure 1). Pre-training tests took place 3 weeks after the end of the amateur league finale (preseason, from April to June 2017). The study orientation, training and testing sessions spanned 12 weeks. During the first 3 weeks (4 days a week), low-intensity exercises were completed for orientation. Before and after the 6 weeks of speed interval training, pre- and post-training tests were performed (in a total of 3 weeks). Temperature, humidity and the altitude of the testing and the training environment were ~18–21°C, % 45–60 and 1285 m respectively.

### Training Protocols

In addition to their routine training (on Monday, Wednesday, Friday) participants from the SIT150 and SIT30 training groups performed running-based 30 s all-out sprints under supervision 2 times per week (on Tuesday and Thursday) (week 1–2: 6 reps; week 3–4: 8 reps; week 5–6: 10 reps). All participants also played a 90-min soccer match among themselves (on Saturday). Before every test and training session, a standard warm-up (5-min jogging, 3-min dynamic stretching [20] and 7-min specific drills) was instructed and supervised by the coach. The recovery intervals between the two bouts for SIT150 and SIT30 training groups were 150 and 30 s respectively. The CG attended solely routine soccer training sessions and 90-min soccer matches. Routine soccer training consisted of low to moderate intensity running based drills with and without a ball (on Monday), small sided games (3 × 3, 4 × 4, 5 × 5, 7 × 7) (on Wednesday), short-distance (5–10 m) maximal sprint, reaction and acceleration training (on Friday).

### Yo-Yo_IRT1_ and Yo-Yo_IRT2_

All subjects were instructed to run between cones placed 20-m apart accordingly with the pre-recorded sound signal and the speed was increased as Bangsbo et al. [21] and Krustrup et al. (2006) stated. After every 40-m run, subjects had 10-s rest in 5-m-long area. The tests were completed when the subject missed two consecutive cones [22]. Although they have the same test settings, the initial running speeds for Yo-Yo_IRT1_ and Yo-Yo_IRT2_ were 10 and 13 km · h^−1^ respectively.

### Graded Treadmill Test

The subjects were instructed to complete a continuous incremental run on treadmill to determine their VO_2max_. The initial speed of the test was 9 km · h^−1^ and was increased 1 km · h^−1^ every minute until reaching the final speed the treadmill allows (16 km · h^−1^). After that, the treadmill grade was increased by 1% every minute until volitional exhaustion. The protocols where treadmill grade was increased by 1% has been shown to provide a valid VO_2max_ measurement [23, 24]. Throughout the tests, expired gas was recorded using Masterscreen metabolic cart (Viasys Healthcare, Jaeger, Würzburg, Germany). The gas analyzers were calibrated as instructed by the manufacturer, with a certified gas mixture of known concentrations. It was considered VO_2max_ if at least two of the following criteria met: the presence of VO_2_ plateau, a respiratory exchange ratio greater than 1.1, and a peak heart rate (HR) greater than 90% of age-predicted maximum (220-age) [25].

### Repeated Sprint Test

Subjects completed twelve 20-m sprints with a 30-s recovery inbetween on a grass field [26]. Newtest (Powertimer 300-series, Finland) telemetric system was used and photocells were placed at 0- and 20-m. Following each sprint, the subjects walked back to the starting line. After the test, the best sprint time (RST_best_), total sprint time (RST_total_; total sprinting time of 12 attempts) and percentage decrement of repeated sprinting times (RST_dec_) were calculated as Glaister et al. [27] suggested.

$$\begin{array}{c} Percentage\;of\;performance\;decrement\\ =(\frac{s1+s2+\ldots +sn}{BST\times n}-1)\times 100 \end{array}$$

where RST_best_ was the best sprint time; s was a given sprint performance (in second), n was the number of the final sprint (in this case 12).

### Statistical Analysis

The study results and descriptive data were reported as mean ± SD. Normality of data was analyzed using Shapiro-Wilk test. Two-way repeated measures ANOVA was used with one within factor (time: pre vs. post) and one between factor (group: SIT150 vs. SIT30 vs. CG). The partial eta squared value (η_p_^2^) was calculated to indicate effect sizes, which can be interpreted as a proportion of variance. Comparing pre- and post-training test results, Paired samples t test (if data was normally distributed) and Wilcoxon test (if data was not normally distributed) were used. Cohen’s d effect sizes were also calculated and the outputs were described as follows: < 0.20 (trivial), 0.20–0.59 (small), 0.6–1.19 (moderate), 1.2–1.99 (large); ≥ 2.0 (very large) [30]. Intraclass correlation coefficient (ICC) with 2-way random model [28] was used to test the consistency of pre & post measurements [29]. Based on 95% confidence interval of the ICC estimate, the values were classified as follows: < 0.5 (poor), 0.5–0.75 (moderate), 0.75–0.9 (good), > 0.90 (excellent). The significance level was set at 0.05 for all tests.

## RESULTS

Inter- and intra- group performances are shown in Table 2 for RSA (RST_best_, RST_total_ and RST_dec_), Yo-Yo_IRT1_, Yo-Yo_IRT2_ and VO_2max_.

**TABLE 2:** Pre- and post-training performance, percentage change and effect size for the sub-groups

	SIT150 n = 8				SIT30 n = 7				CG n = 8			
	Pre	Post	d	%Ch	Pre	Post	d	%Ch	Pre	Post	d	%Ch
RST_best_ (s)	2.8 ± 0.1	2.8 ± 0.1	0	0	2.8 ± 0.1	2.9 ± 0.8*	0.17 trivial	↑3.5	2.9 ± 0.1	2.9 ± 0.1	0	0

RST_total_ (s)	35.8 ± 7.1	35.6 ± 11.9	0.02 trivial	↓0.5	35.5 ± 1.7	36.4 ± 1.1*	0.74 moderate	↑2.5	36.6 ± 1.6	36.4 ± 1.8	0.11 trivial	↓0.5

RST_dec_ (s)	4.3 ± 2.2	3.7 ± 1.9	0.29 small	↓13.9	3.6 ± 2.4	3.7 ± 1.2*	0.05 trivial	↑3.7	5.1 ± 2.3	4.1 ± 2.2	0.44 small	↓24.3

Yo-Yo_IRT1_ (m)	1360 ± 389.5	1945 ± 397*	1.48 large	↑43	1531 ± 411	1885 ± 512*	0.76 moderate	↑23.1	1445 ± 493	1735 ± 524*	0.57 small	↑20

Yo-Yo_IRT2_ (m)	625 ± 135.1	830± 187.3*	1.25 large	↑32.8	751 ± 165	948 ± 141*	1.28 large	↑26.2	630 ± 262	750 ± 247	0.47 Small	↑19

VO_2max_ (ml · kg^−1^ · min^−1^)	42.7 ± 5.3	50.4 ± 4.5*	1.56 large	↑18	42.4 ± 3.4	50.5 ± 4.7*	1.97 large	↑19.1	42.4 ± 8.3	48.1 ± 6.8*	0.75 moderate	↑13.4

Data presented as mean± SD. RST_best;_ best sprint time; RST_total_: total sprint time; RST_dec_: the percentage of performance decrement; Yo-Yo_IRT1_: Intermittent Recovery Test level 1; Yo-Yo_IRT2_: Intermittent Recovery Test level 2; VO_2max_: maximal oxygen uptake; (d): Cohen’s d effect size; %Ch: percentage change.

* Significant difference between pre- and post-training

**FIG. 2:**
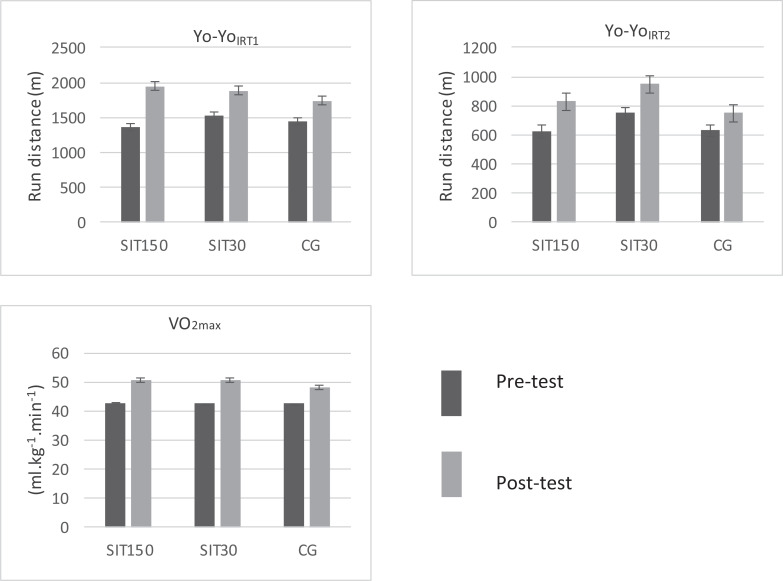
Baseline and post training Yo-Yo_IRT1_, Yo-Yo_IRT2_ and VO_2max_ performance level of SIT150, SIT30 and CG.

The two-way repeated measures ANOVA results revealed that there was a significant main effect of time (pre- post) for Yo-Yo_IRT1_ (p = 0.001, η_p_^2^ = 0.88), Yo-Yo_IRT2_ (p = 0.01, η_p_^2^ = 0.70) and VO_2max_ (p = 0.001, η_p_^2^ = 0.84); and no significant main effect of time or group (S150, SIT30, CG) for RST_best_, RST_total_ and RST_dec_ (p > 0.05). There was no time*group interaction effect for any variable (Figure 2).

Intra-group comparisons indicated significant differences. A significant performance decrement in RST_best_ (3.5%, p = 0.029, Cohen’s d = 0.17) and RST_total_ (2.5%, p = 0.038, Cohen’s d = 0.74) was present in the SIT30 training group, whereas no significant difference in RST_dec_ was found (p > 0.05). In the SIT150 training group, RST_best_ did not significantly change (p > 0.05), similarly, no statistically significant change was present in RST_total_ (0.5%) and RST_dec_ (13.9%), nonetheless, there were relative improvements. Although there was no significant difference in RST_best_, RST_total_ and RST_dec_, performance improvement in CG was seen in RST_dec_ (24.3%, p > 0.05).

A greater improvement was seen in Yo-Yo_IRT1_ performance in SIT150 group (43%, p = 0.001, Cohen’s d = 1.48) compared to SIT30 (23%, p = 0.002, Cohen’s d = 0.76) and CG (20%, p = 0.013, Cohen’s d = 0.57).

There was a significant improvement in Yo-Yo_IRT2_ performance in SIT30 (26.2%, p = 0.018, Cohen’s d = 1.28) and even a greater improvement in SIT150 (32.8%, p = 0.008, Cohen’s d = 1.25) training group.

VO_2max_ (ml · kg^−1^ · min^−1^) significantly changed in all groups (SIT150 [18%, p = 0.010, Cohen’s d = 1.56], SIT30 [19.1%, p = 0.001, Cohen’s d = 1.97], and CG [13.4%, p = 0.002, Cohen’s d = 0.75]).

ICC for pre- and post- tests was as follows: SIT30 VO_2max_ (.802, p = 0.035), Yo-Yo_IRT1_ (.958, p = 0.001), Yo-Yo_IRT2_ (.890, p = 0.008), RST_best_ (.857, p = 0.016), RST_total_ (.890, p = 0.008), and RST_dec_ (.403, p = 0.273); SIT150 VO_2max_ (.334, p = 0.303), Yo-Yo_IRT1_ (.822, p = 0.018), Yo-Yo_IRT2_ (.696, p = 0.070), RST_best_ (.0, p = 0.926), RST_total_ (.0, p = 0.648), and RST_dec_ (.131, p = 0.429); CG VO_2max_ (.948, p = 0.001), Yo-Yo_IRT1_ (.936, p = 0.001), Yo-Yo_IRT2_ (.522, p = 0.176), RST_best_ (.941, p = 0.001), RST_total_ (.796, p = 0.026), and RST_dec_ (.736, p = 0.05) (Table 3).

**TABLE 3:** Pre and post test conditions Intraclass Correlation Coefficients for VO_2max_, Yo-Yo_IRT1_, Yo-Yo_IRT2_, RST_best_, RST_total_, and RST_dec_.

	SIT30		SIT150		CG	

	ICC	p	ICC	p	ICC	p
VO_2max_	.802	.035	.334	.303	.948	.001
Yo-Yo_IRT1_	.958	.001	.822	.018	.936	.001
Yo-Yo_IRT2_	.890	.008	.696	.070	.522	.176
RST_best_	.857	.016	.0	.926	.941	.001
RST_total_	.890	.008	.0	.648	.796	.026
RST_dec_	.403	.273	.131	.429	.736	.05

## DISCUSSION

In this study, the performance adaptations of soccer players to 2 variations of 6 weeks running-based SIT protocols with different recovery intervals were investigated. The main findings of the study indicated that both SIT150 and SIT30 training methods improve Yo-Yo_IRT1_, Yo-Yo_IRT2_ and VO_2max_ performance. However, SIT150 training appeared to ensure greater performance improvements.

The structure of Yo-Yo_IRT1_ is known to fit the soccer game profile [31]. Previous research showed that HI training enhances Yo-Yo_IRT1_ performance [14, 32, 33, 34]. Nyberg et al., [32] investigated the effects of 9 weeks HIIT interval training (30 m all-out runs, 8–10 reps with 10 s rest in between; 3 sets with 3 minutes recovery intervals) in 13 semi-professional male soccer players during league season and reported that after the training period Yo-Yo_IRT1_ performance was improved by 11.6%. Hill-Hass et al. [33]. showed that 7 weeks of speed endurance training enhanced Yo-Yo_IRT1_ performance by 22.1% whereas Thomassen et al. [14] reported a 6.1% improvement in Yo-Yo_IRT1_ performance after 4 weeks of training (10–12 reps. × 30-s all-out running, 3 days a week). Our results were similar to the findings of previous investigations (Yo-Yo_IRT1_ improved by 43% in SIT150 and 23% in SIT30). The SIT30 training has shorter recovery intervals compared to the SIT150 training method, causing the SIT30 method to rely more on the anaerobic pathways for exercise metabolism. As supported by the previous research and the findings of this study, SIT150 training may be considered as a convenient method to enhance oxidative systems during a soccer match. High intensity interval training (HIIT) with relatively longer recovery bouts seemed to stimulate intra-muscle adaptations and produce a better quality training period compared to same training with shorter recovery intervals. Consequently, this method can be used in soccer players to enhance their capacity to perform high-intensity exercises.

In a study conducted by Mohr & Krustrup [15] on 18 sub-elite soccer players, 4 weeks of HIIT induced improvements in Yo-Yo_IRT2_ performance of training group-1 (W:R; 1:1) (26%), and even greater improvements training group-2 (W:R; 1:5) (50%). Authors also emphasized that having longer recovery periods after a high-intensity exercise, SIT150 training may have improved the fatigue resistance by stimulating a wide range of muscular system adaptations. In a similar study by Iaia et al. [35] 13 soccer players were trained for 3 weeks with 3 sessions per week. The observed performance improvements of Yo-Yo_IRT2_ were greater in group-1 (6–8 reps × 20 s with 120 s recovery intervals) (10%, p < 0.001) compared to group-2 (6–8 reps × 20 s with 40 s recovery intervals) (4%, p < 0.049). Also, it was highlighted that Yo-Yo_IRT2_ performance improvements were more likely to associate with the ability of the muscle to produce forceful bouts rather than enhancing exercise tolerance [36]. While, no studies were investigating the effects of HI training at short and long recovery intervals that were utilized in this study (*i.e.* 150-s & 30), our results were in line with the observations reported in the literature. Moreover, the present study showed that the SIT150 improved the capacity to perform high-intensity exercises (improvements in Yo-Yo_IRT2_ performance by 32%). It should be noted that considering the Yo-Yo_IRT2_ and soccer have a similar nature (*i.e.* HI running bouts, change of direction & intermittent nature), SIT150 training may provide great performance gains in soccer.

Both the total distance covered and the distance covered at HI runs are particularly important in soccer. It was shown that Yo-Yo_IRT1_ results correlated with the total distance covered in a soccer match [36] whereas Yo-Yo_IRT2_ performance correlated with distance covered at HI [37]. Consequently, both these tests reflect various aspects of fitness level [38].

SIT protocols with different recovery periods elicited similar increase in VO_2max_ [11, 13]. Olek et al. [11] reported similar VO_2max_ improvements after 2 weeks SIT period (3 sessions a week, 6 sessions in total) in two different SIT training groups (W:R; 10:60 s; 10:240 s). Similarly, Hazel et al. [13] showed that 2-weeks SIT protocols with different recovery intervals (10 s all-out run with either 120 s or 240 s rest) induced similar increase in VO_2max_. The results of the present study showed similar VO_2max_ improvements both in SIT150 and SIT30 training groups. As a result, to enhance VO_2max_ in amateur soccer players, either one of these methods may be utilized. Common finding of these studies indicate that the change in the duration of the recovery period may not be the key variable to get better VO_2max_ gains after VHI training sessions.

Mohr & Krustrup [15] reported that 4 weeks of HIIT improved the RST_best_ (1.7%), RST_dec_ (4.4%) and RST_total_ (2.1%) in SIT group (30 s all-out running, 8–10 repetitions with a passive rest period of 150 s in between). Iaia et al. [35], investigating the effects of HIIT on RSA, reported that group-1 (20 s all-out running with a rest period of 120 s; 6–8 repetitions) had improvements (%2.5), while there were no changes in group-1 (20 s all-out running with a rest period of 40 s; 6–8 repetitions). Furthermore, Ingebrigtsen et al. [39] showed that RSA was not improved significantly after 6 weeks of HIIT in young elite soccer players. In our study, the RST_dec_ (13.9%) and RST_total_ (0.5%) were improved in SIT150 and were impaired in SIT30 training group.

A limitation of this study is the control group which did not match with the total volume of the training groups. Most studies did not use a control group making it difficult to understand the amplitude of the change induced by speed endurance training. Given that these investigations tested the effects of additional training combined with routine training, it was not clear what caused the changes in performance. Another limitation of the present study was that the graded treadmill test protocol was not used in the literature. The maximum speed that the treadmill could reach was 16 km · h^−1^. To raise the intensity of the test we increased the treadmill grade by 1% every minute. Additionally, in this study design we did not control the work-load, since the players were not used to any workload monitoring system; however, we have tried our best to ensure that both training interventions resulted in a consistent amount of sprints performed by the players of both groups. We are aware that the difference in recovery time in-between sprints would potentially result in a difference in overall sessions’ training load, making this a weakness of the present study. Therefore, further studies with similar protocols should consider monitoring training load. Lastly, the population of the present study was limited to only 23 amateur soccer players.

## CONCLUSIONS

This study was the first to investigate two SIT protocols with different work-rest ratios (1:5 & 1:1) in amateur soccer players. Both SIT protocols were effective in improving Yo-Yo_IRT1_, Yo-Yo_IRT2_ and VO_2max_ performance. However, SIT150 training (30 s all-out sprints with 150 s recovery intervals; 6 to 10 reps; for 6 weeks) induced greater improvements likely due to having longer recovery intervals between repetitions, and can be used as a training method to improve Yo-Yo_IRT1_, Yo-Yo_IRT2_ and VO_2max_ performance of amateur soccer players. Such a method may be particularly useful for amateur soccer players to improve a range of performance characteristics.
